# Radiomic subtypes predict survival and chemotherapy benefit in stage I lung adenocarcinoma: a multicenter study

**DOI:** 10.1186/s13244-026-02228-1

**Published:** 2026-03-02

**Authors:** Guangyu Tao, Dongying Wang, Xin Cheng, Zhenghai Lu, Hua Zhong, Hong Yu, Wei Nie

**Affiliations:** 1 Department of Radiology, Shanghai Chest Hospital, Shanghai Jiao Tong University School of Medicine, Shanghai, China; 2 Department of Respiratory and Critical Care Medicine, Shanghai Chest Hospital, Shanghai Jiao Tong University School of Medicine, Shanghai, China; 3 Shanghai Key Laboratory of Thoracic Tumor Biotherapy, Shanghai Chest Hospital, Shanghai Jiao Tong University School of Medicine, Shanghai, China; 4 Shanghai University of Medicine & Health Sciences, Shanghai, China; 5 Department of Radiology, Xinhua Hospital, Shanghai Jiao Tong University School of Medicine, Shanghai, China

**Keywords:** Lung adenocarcinoma, Radiomics, Unsupervised clustering, Prognosis, Adjuvant chemotherapy

## Abstract

**Objectives:**

Postoperative survival outcomes vary substantially among patients diagnosed with stage I lung adenocarcinoma (LUAD). This study aimed to develop CT-based radiomic subtypes using unsupervised clustering to assess their association with overall survival (OS), systemic nutritional-inflammatory status, and adjuvant chemotherapy benefit.

**Materials and methods:**

A total of 496 stage I LUAD patients from two independent centers were included. Preoperative CT radiomic features (*n* = 1218) were extracted, and subtypes were derived using the K-means clustering algorithm. The independent prognostic value of these subtypes, along with their capacity to predict the benefit of adjuvant chemotherapy, was evaluated through multivariable Cox regression and treatment-by-subtype interaction analyses.

**Results:**

Three radiomic subtypes with significant prognostic differences in OS were identified. The high-risk subtype, Cluster 2, exhibited distinct clinical characteristics and was associated with markedly poorer OS (hazard ratio [HR] = 15.71, *p* < 0.001, compared to Cluster 0). Cluster 2 also showed an inflammatory imbalance, with elevated systemic immune-inflammation index and neutrophil-to-lymphocyte ratio, and  a decreased lymphocyte-to-monocyte ratio. Notably, a significant interaction was found between subtypes and adjuvant chemotherapy (interaction *p* < 0.001, Cluster 2 vs Cluster 0). Subgroup analysis indicated that stage IB patients within Cluster 2 derived a significant survival benefit from adjuvant chemotherapy (interaction *p* = 0.003 vs Cluster 0).

**Conclusions:**

This study developed a CT-based radiomic subtype system using unsupervised clustering that identifies high-risk stage I LUAD patients with systemic inflammatory imbalance. Notably, these subtypes predict differential survival benefits from adjuvant chemotherapy in high-risk stage IB patients, thereby supporting personalized postoperative treatment strategies.

**Critical relevance statement:**

This CT-based radiomic subtype system stratifies prognosis and identifies stage I LUAD patients who may benefit from adjuvant chemotherapy, enabling personalized treatment decisions in radiology.

**Key Points:**

- Conventional tumor-node-metastasis (TNM) staging does not adequately capture tumor heterogeneity in stage I LUAD.
- Three CT-based radiomic subtypes were established, with the high-risk subgroup correlating with systemic inflammatory imbalance and poorer OS.
- CT-based radiomic stratification identifies stage IB patients who benefit from adjuvant chemotherapy, supporting personalized postoperative management.

**Graphical Abstract:**

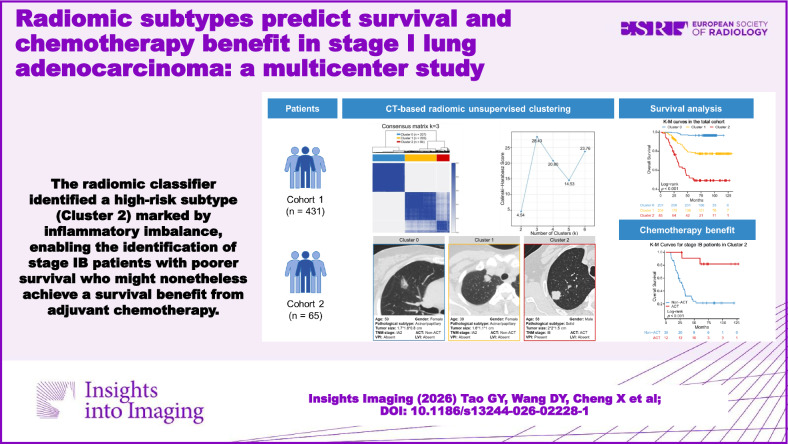

## Introduction

Lung adenocarcinoma (LUAD), the most prevalent lung cancer subtype worldwide [1], represents approximately 50% of all lung cancer cases [2, 3]. While surgery is the primary curative option for early-stage LUAD [4, 5], survival outcomes vary significantly following resection. Data from the Japanese Joint Committee of Lung Cancer Registry show a decline in 5-year survival rates across stage I subtypes, with stage IB patients having a rate of just 71.5%, indicating a relatively poor prognosis [6]. Postoperative adjuvant chemotherapy is advised for stage IB patients exhibiting high-risk pathological features, such as poor differentiation, vascular invasion, or visceral pleural invasion (VPI) [7, 8]. However, this tumor-node-metastasis (TNM) staging system-based approach does not  adequately address tumor heterogeneity, limiting accurate risk assessment. Thus, there is an urgent need for a more precise prognostic tool to enhance postoperative management and optimize adjuvant treatment strategies.

In recent years, computed tomography (CT)-derived radiomic features have emerged as a valuable tool for the diagnosis and prognostic assessment of lung cancer [9–11]. We previously developed a radiomic model using supervised learning, which proved effective for risk stratification and predicting the survival benefits of adjuvant chemotherapy in patients with stage I LUAD [12]. Nonetheless, these methodologies are often limited by their reliance on predefined labels and reduced interpretability, which constrains their capacity to reveal novel subtypes or identify potential biological heterogeneity. In contrast, unsupervised learning techniques, such as clustering analysis, offer the potential to discern radiomic subtypes associated with prognosis and the tumor microenvironment without requiring predefined labels, thereby effectively addressing these constraints [13]. Among available unsupervised algorithms, K-means clustering was chosen in this study. It provides a relatively simple and interpretable partition of patients into discrete subgroups based on radiomic feature patterns, has good reproducibility when applied to standardized features, and has been successfully used in previous radiomics studies for subtype discovery, outcome stratification, and identification of subgroups associated with specific molecular and pathological features. For example, Perez-Johnston et al employed consensus clustering on preoperative CT images from patients with stage I LUAD, identifying four distinct radiomic subtypes that demonstrated significant differences in survival outcomes [14]. Notably, the high-risk group was further characterized by aggressive molecular features, including STK11 mutations and activation of the PI3K pathway. Similarly, Wen et al confirmed that radiomic features of stage IA LUAD could reflect different tumor biological behaviors, such as invasiveness, occult lymph node metastasis, and histological grade [15].

Building on these observations that radiomic subtypes can reflect intrinsic tumor biology, we further sought to investigate how systemic host factors might biologically contextualize imaging-derived risk groups. An increasing body of evidence indicates that nutritional status and cancer-related inflammatory responses are closely linked to disease progression and survival outcomes [16–18]. Characterizing systemic nutritional-inflammatory indices across radiomic subtypes may therefore provide a biological context for imaging-derived risk groups, helping to explain why certain subtypes are associated with unfavorable outcomes. However, the relationship between radiomic subtypes and systemic nutritional-inflammatory status remains underexplored. In addition, the ability of radiomic subtypes to predict the responses to adjuvant chemotherapy is not yet well understood. Therefore, this study aims to develop a radiomic stratification system based on unsupervised clustering to identify high-risk patients with stage I LUAD and to examine the association of these subtypes with systemic nutritional-inflammatory indices. Furthermore, the study evaluates the differential responses to adjuvant chemotherapy among subgroups to more effectively inform individualized treatment strategies.

## Materials and methods

### Study design and patient population

This study received approval from the Ethics Committees of Shanghai Chest Hospital (approval number KS1596, dated August 28, 2019) and Xinhua Hospital (approval number XHECD-2021-137, dated October 18, 2021). The requirement for written informed consent was waived due to the use of de-identified data. The study adhered to the 9th edition of the International Association for the Study of Lung Cancer (IASLC) TNM classification to identify patients with stage I LUAD [19, 20]. The exclusion criteria for surgical LUAD patients included: (1) presence of multiple primary LUADs; (2) no lobectomy performed; (3) diagnosis of adenocarcinoma in situ (AIS) or microinvasive adenocarcinoma (MIA); (4) lack of preoperative high-resolution CT (HRCT) images obtained within two weeks prior to surgery; (5) death within one month after surgery; and (6) loss to follow-up. A total of 496 patients were included in the study, comprising 431 patients treated at Shanghai Chest Hospital between March 2009 and May 2016, and 65 patients treated at Xinhua Hospital between February 2015 and March 2021. The detailed screening criteria and patient recruitment process for both cohorts are presented in Fig. S1.

### Clinicopathological characteristics

In this study, the clinicopathological variables collected included age, gender, smoking history, tumor location, pathological subtype, tumor size, TNM stage, VPI, and lymphovascular invasion (LVI). The histological subtypes of LUAD were classified according to the IASLC/American Thoracic Society (ATS)/European Respiratory Society (ERS) criteria [21]. Tumor staging was based on the 9th edition IASLC lung cancer staging classification [19, 20]. Peripheral blood-based nutritional and inflammatory markers included the prognostic nutritional index (PNI), systemic immune-inflammation index (SII), lymphocyte-to-monocyte ratio (LMR), and neutrophil-to-lymphocyte ratio (NLR). PNI was calculated as follows: PNI = albumin (g/L) + 5 × lymphocyte count (10^9^/L). Inflammation-based indices were calculated as follows: SII = platelets × neutrophils/lymphocytes; LMR = lymphocytes/monocytes; and NLR = neutrophils/lymphocytes [22].

### Follow-up protocol and clinical outcome assessment

All patients were followed up every 3 months during the first 2 years and every 6 months thereafter. Clinical assessments included physical examinations, blood tests, chest CT, abdominal CT, or ultrasound. Additionally, annual whole-body bone scans and head CT or magnetic resonance imaging were conducted. In this study, the primary outcome was overall survival (OS), defined as the time from radical surgery to death from any cause.

### CT data acquisition and radiomic feature detection

All scans were acquired during full inspiration under breath-hold conditions with patients in a supine position. Non-contrast chest CT examinations were performed using one of four scanner models: Discovery CT750HD (GE Healthcare), Revolution CT (256-detector row, GE Healthcare), Brilliance (64-detector row, Philips Healthcare), and uCT S160 (16-detector row, United Imaging). HRCT protocols were applied with the following parameters: detector collimation width of 0.625–1.25 mm, pitch factor of 0.64, reconstructed slice thickness of 0.625–1.25 mm without gap, matrix size of 512 × 512 or 1024 × 1024, field of view (FOV) ranging from 350 to 400 mm, tube voltage of 120 kVp, and tube current between 220 and 300 mA. All images used for radiomic analysis were reconstructed directly on the scanner consoles from the original raw data using vendor-provided standard high-resolution lung algorithms in accordance with the routine clinical HRCT protocols at each institution. No additional retrospective reconstruction, kernel modification, or offline post-processing was performed specifically for this study.

Tumor regions of interest (ROIs) were manually delineated at the voxel level on preoperative CT images by a radiologist (10 years of experience in thoracic CT) using 3D Slicer software (v4.8.0, Brigham and Women’s Hospital, USA). These segmentations were subsequently reviewed and verified by a second senior radiologist (30 years of experience in thoracic imaging).

Radiomic feature extraction was performed using the AK platform (Analysis Kit, v3.3.0, GE Healthcare), which is compliant with the Image Biomarker Standardization Initiative (IBSI). In total, 1218 quantitative features were extracted, including first-order intensity statistics, shape-based descriptors, and second-order texture features derived from gray-level co-occurrence, run-length, size-zone, and dependence matrices, as well as higher-order features obtained after wavelet and Laplacian of Gaussian transformations. During feature extraction, voxel dimensions (in-plane pixel spacing and slice thickness) were obtained from the DICOM data and used to calculate features in physical space, so that CT scans with matrix sizes of 512 × 512 or 1024 × 1024 were analyzed at their native resolution without additional up- or down-sampling.

### Radiomic feature preprocessing and clustering model selection

This study included a total of 1218 radiomic features. To account for differences in feature scales, all features were standardized using *z*-score normalization before clustering. For the clustering process, we evaluated two widely recognized algorithms: K-means with k-means++ initialization, constrained to a maximum of 100 iterations, and DBSCAN, which involved tuning the eps parameter using the k-distance method and a minimum of six samples. A multi-criteria framework was employed to evaluate the rationality and stability of the feature space partitioning achieved by these models. Visual inspection of the consensus matrix indicated that the clustering performance of K-means was markedly superior to that of DBSCAN. Notably, the consensus matrix derived from K-means demonstrated high intra-cluster co-clustering frequencies and distinct block-diagonal patterns. As a result, K-means was identified as the optimal clustering method for subsequent analyses.

### Optimal cluster number determination and stability validation

The optimal number of clusters for the K-means algorithm was determined using the Calinski–Harabasz (CH) index and the silhouette coefficient. Cluster stability was then evaluated by performing 30 independent K-means runs with different random initializations. Reproducibility was quantified using a clustering consistency *R*² and the mean Adjusted Rand Index (ARI) across runs, and was compared with ARI values obtained from random label permutations.

### Statistical analysis

Continuous variables were summarized as medians with interquartile ranges (IQR). For comparisons of continuous variables, including age and nutritional-inflammatory indices (PNI, SII, LMR, and NLR), across the three radiomic subtypes, global differences were first assessed using the Kruskal–Wallis test. When the global test was statistically significant, post-hoc pairwise Wilcoxon rank-sum tests were performed between clusters with Bonferroni correction for the three pairwise comparisons. Categorical variables were described as counts and percentages and compared across radiomic subtypes using the χ² test or Fisher’s exact test, as appropriate. Kaplan–Meier survival curves and log-rank tests were utilized to evaluate survival differences between clusters. Multivariable Cox proportional hazards models were employed to estimate hazard ratios (HR) along with 95% confidence intervals (CI). All statistical analyses and graphical representations were performed using R version 4.4.1 (R Foundation for Statistical Computing) and IBM SPSS Statistics version 26.0 (IBM Corp., Armonk), with a two-sided *p* value of less than 0.05 considered statistically significant.

## Results

### Baseline characteristics of patients

This study comprised 496 patients with LUAD from two centers. The median age of the cohort was 60 years, with 228 individuals (46.0%) being male. A significant proportion of the patients, 424 (85.5%), were non-smokers. The majority of patients were classified within the acinar/papillary pathological subgroup [*n* = 408 (82.3%)], while 67 patients (13.5%) were identified as lepidic, and 21 patients (4.2%) were categorized as solid/micropapillary. Tumor sizes were distributed as follows: 19.0% of patients had tumors measuring 0–1 cm, 45.4% had tumors measuring 1–2 cm, 26.4% had tumors measuring 2–3 cm, and 9.3% had tumors measuring 3–4 cm. Regarding cancer staging, 383 patients (77.2%) were classified as stage IA, and 113 patients (22.8%) as stage IB. Among the entire cohort, 86 patients (17.3%) exhibited VPI, and 13 patients (2.6%) demonstrated LVI. Additionally, 60 patients (12.1%) underwent adjuvant chemotherapy. Adjuvant chemotherapy regimens were retrieved from the electronic medical record systems of both centers and consisted of platinum-based doublet chemotherapy, with cisplatin or carboplatin combined with vinorelbine, paclitaxel, gemcitabine, or pemetrexed. Baseline clinicopathological characteristics according to the three radiomic subtypes are summarized in Table 1, and Bonferroni-corrected *p* values from pairwise comparisons between clusters (Cluster 1 vs Cluster 0, Cluster 2 vs Cluster 0, and Cluster 2 vs Cluster 1) are provided in Supplementary Table S1.

**Table 1:** Baseline characteristics of patients

Characteristic	Total (*n* = 496)	Cluster 0 (*n* = 207)	Cluster 1 (*n* = 204)	Cluster 2 (*n* = 85)	*p* value
Age (years)	60 [53, 67]	60 [54, 67]	59 [51, 66]	63 [55, 68]	0.015
Gender					0.003
Male	228 (46.0)	80 (38.6)	97 (47.5)	51 (60.0)	
Female	268 (54.0)	127 (61.4)	107 (52.5)	34 (40.0)	
Smoking history					< 0.001
Never	424 (85.5)	186 (89.9)	181 (88.7)	57 (67.1)	
Ever	72 (14.5)	21 (10.1)	23 (11.3)	28 (32.9)	
Tumor location					0.827
Upper left	99 (20.0)	39 (18.8)	43 (21.1)	17 (20.0)	
Lower left	77 (15.5)	26 (12.6)	35 (17.2)	16 (18.8)	
Upper right	175 (35.3)	79 (38.2)	65 (31.9)	31 (36.5)	
Right middle	47 (9.5)	20 (9.7)	20 (9.8)	7 (8.2)	
Lower right	98 (19.8)	43 (20.8)	41 (20.1)	14 (16.5)	
Pathological subtype					< 0.001
Lepidic	67 (13.5)	19 (9.2)	42 (20.6)	6 (7.1)	
Acinar/papillary	408 (82.3)	186 (89.9)	150 (73.5)	72 (84.7)	
Solid/micropapillary	21 (4.2)	2 (1.0)	12 (5.9)	7 (8.2)	
Tumor size (cm)					< 0.001
0–1	94 (19.0)	31 (15.0)	59 (28.9)	4 (4.7)	
1–2	225 (45.4)	101 (48.8)	111 (54.4)	13 (15.3)	
2–3	131 (26.4)	59 (28.5)	34 (16.7)	38 (44.7)	
3–4	46 (9.3)	16 (7.7)	0 (0.0)	30 (35.3)	
TNM stage					< 0.001
IA	383 (77.2)	168 (81.2)	181 (88.7)	34 (40.0)	
IB	113 (22.8)	39 (18.8)	23 (11.3)	51 (60.0)	
VPI					< 0.001
Absent	410 (82.7)	180 (87.0)	178 (87.3)	52 (61.2)	
Present	86 (17.3)	27 (13.0)	26 (12.7)	33 (38.8)	
LVI					0.026
Absent	483 (97.4)	197 (95.2)	201 (98.5)	85 (100.0)	
Present	13 (2.6)	10 (4.8)	3 (1.5)	0 (0.0)	
ACT					0.038
Non-ACT	436 (87.9)	183 (88.4)	185 (90.7)	68 (80.0)	
ACT	60 (12.1)	24 (11.6)	19 (9.3)	17 (20.0)	

Data are presented as median [IQR] or *n* (%)

*TNM stage* tumor-node-metastasis stage, *VPI* visceral pleural invasion, *LVI* lymphovascular invasion, *ACT* adjuvant chemotherapy, *IQR* interquartile range

### Three radiomic subtypes of LUAD

**Fig. 1:**
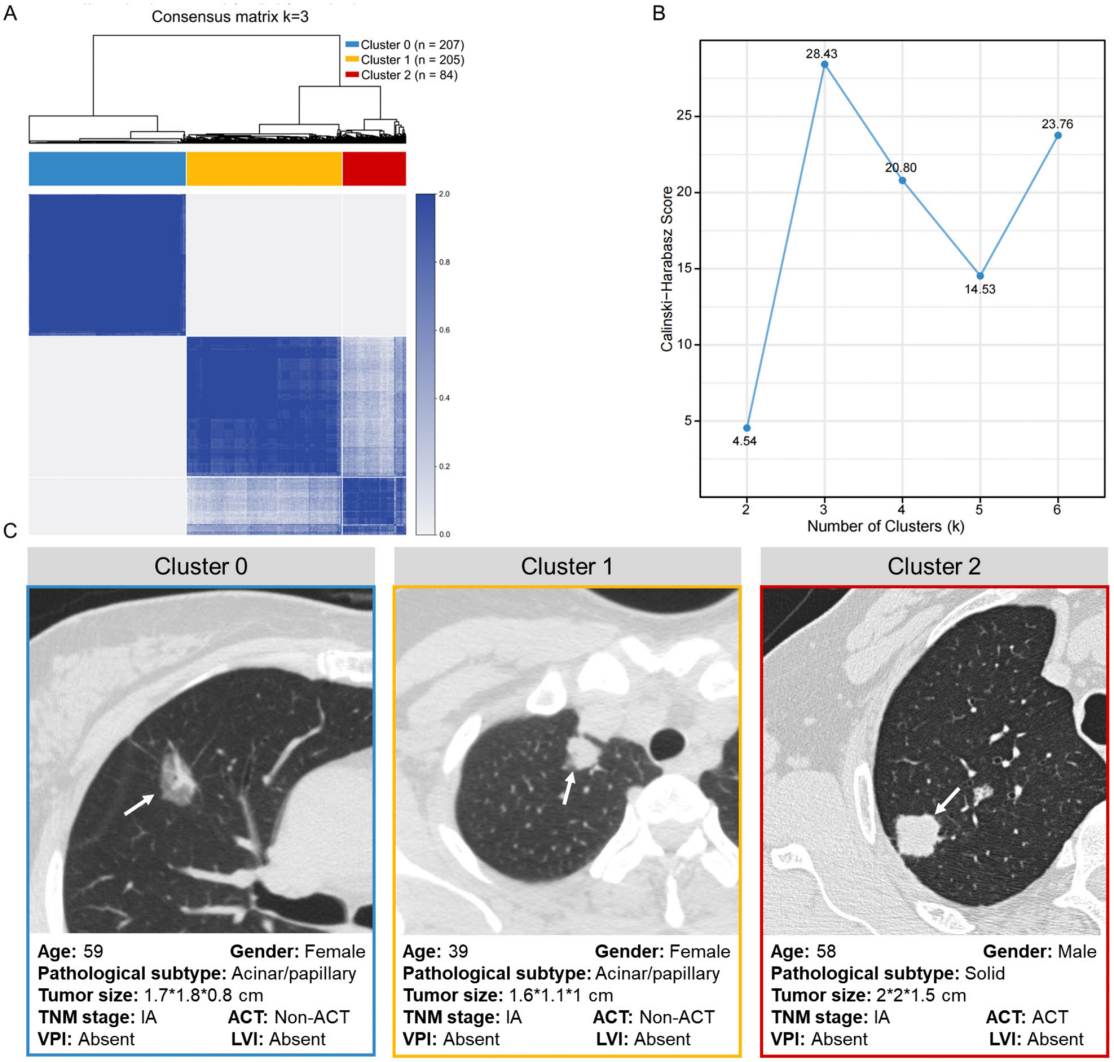
Three radiomic subtypes of LUAD. **A** K-means-based consensus matrix heat map (*k* = 3) depicting consensus values. **B** Calinski–Harabasz index for different cluster numbers. **C** Representative CT images and corresponding clinicopathological characteristics for each cluster. TNM stage, tumor-node-metastasis stage; ACT, adjuvant chemotherapy; VPI, visceral pleural invasion; LVI, lymphovascular invasion

**Fig. 2:**
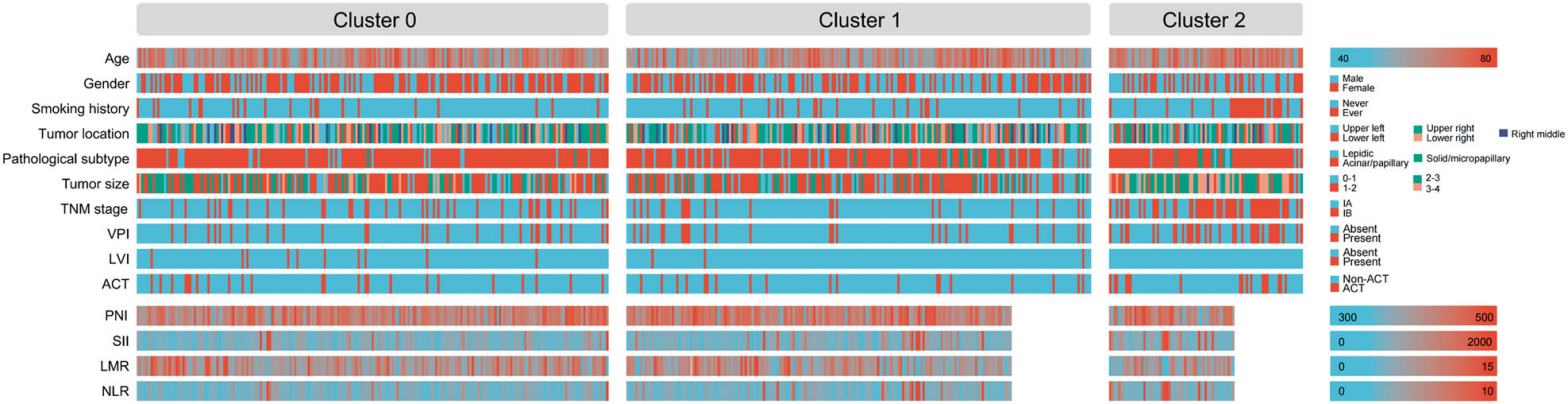
Baseline characteristics of patients

A K-means clustering analysis identified an optimal number of three clusters (*k* = 3), which effectively partitioned the entire cohort into three distinct radiomic subtypes (Fig. 1A, B). The three-cluster solution also showed excellent stability, with a clustering consistency *R*² of 0.9978 and a mean ARI of 0.9978 (standard deviation 0.0025), whereas random label permutations yielded ARI values close to 0, supporting the high reproducibility of the identified radiomic subtypes. Figure 1C illustrates the representative CT imaging and clinicopathological characteristics associated with each of the three clusters. Cluster 0 comprised 207 patients (41.7%), Cluster 1 comprised 204 patients (41.1%), and Cluster 2 comprised 85 patients (17.1%) (Table 1 and Fig. 2). Compared to the other clusters, Cluster 2 had a significantly older median age (median: 63 years; *p* = 0.015). Notably, Cluster 2 exhibited a higher proportion of men [*n* = 51 (60.0%); *p* = 0.003], smokers [*n* = 28 (32.9%); *p* < 0.001], and individuals with solid/micropapillary histology [*n* = 7 (8.2%); *p* < 0.001]. Additionally, this cluster was characterized by a greater prevalence of tumors measuring 2–4 cm [*n* = 68 (80.0%); *p* < 0.001], stage IB disease [*n* = 51 (60.0%); *p* < 0.001], and VPI [*n* = 33 (38.8%); *p* < 0.001]. Furthermore, a significantly higher proportion of patients in Cluster 2 received adjuvant chemotherapy compared to the other clusters [*n* = 17 (20.0%); *p* = 0.038].

### Radiomic subtypes as independent predictors of OS

**Fig. 3:**
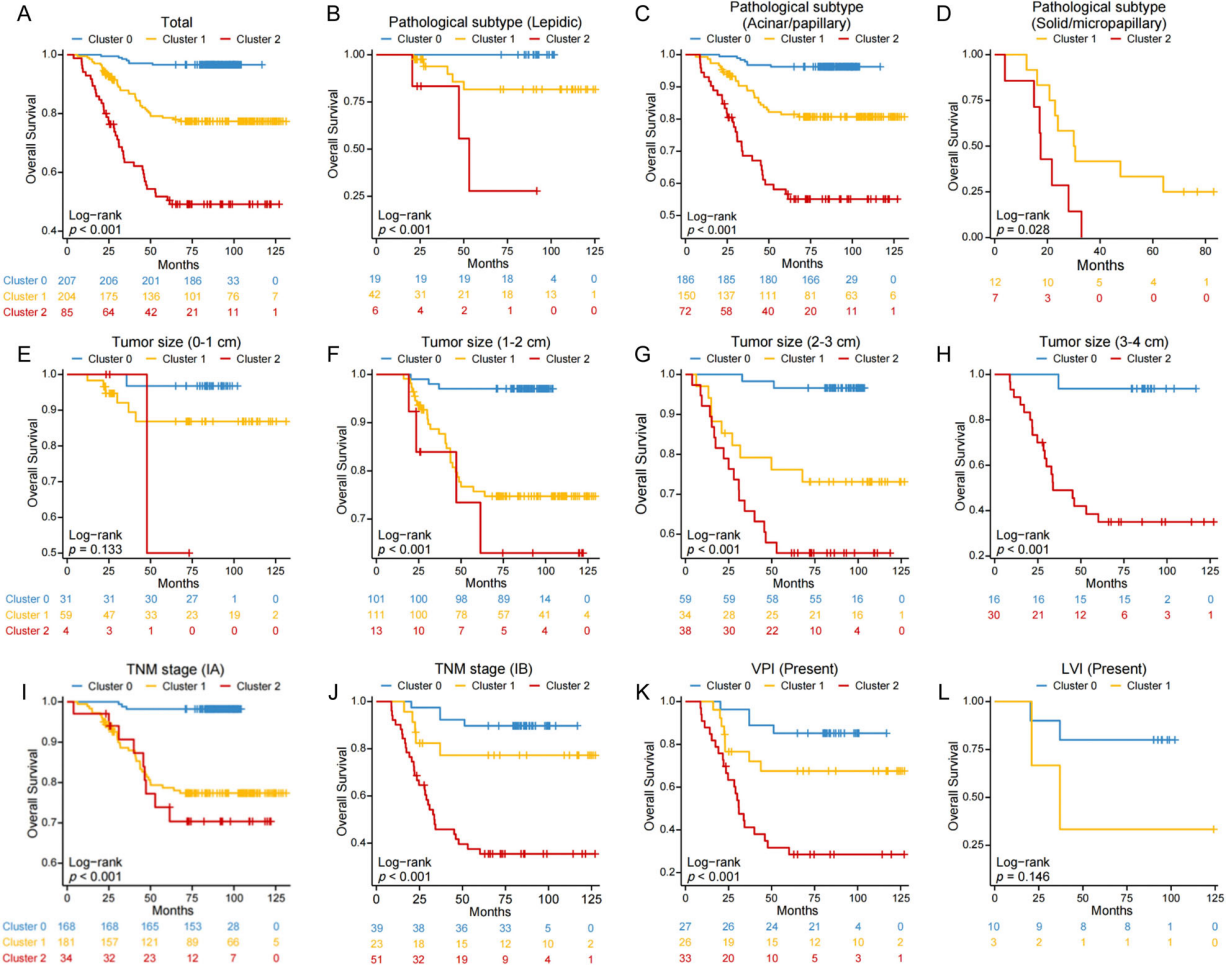
Kaplan–Meier OS curves according to the radiomic subtypes in the total cohort and various patient subgroups. **A** The Kaplan–Meier survival curves compare OS between the three Clusters in the total cohort (*p* < 0.001). **B**–**D** The Kaplan–Meier survival curves compare OS between the three Clusters in patients with lepidic (*p* < 0.001), acinar/papillary (*p* < 0.001), solid/micropapillary (*p* = 0.028). **E**–**H** The Kaplan–Meier survival curves compare OS between the three clusters in patients with tumor sizes of 0–1 cm (*p* = 0.133), 1–2 cm (*p* < 0.001), 2–3 cm (*p* < 0.001), 3–4 cm (*p* < 0.001). **I**–**J** The Kaplan–Meier survival curves compare OS between the three clusters in patients with stage IA (*p* < 0.001), stage IB (*p* < 0.001). **K**, **L** The Kaplan–Meier survival curves compare OS between the three clusters in patients with VPI (*p* < 0.001), LVI (*p* = 0.146). OS, overall survival; TNM stage, tumor-node-metastasis stage; VPI, visceral pleural invasion; LVI, lymphovascular invasion

The Kaplan–Meier analysis, accompanied by log-rank tests, demonstrated statistically significant differences in OS among the three clusters (*p* < 0.001; Fig. 3A). These survival differences were consistently observed across pathological subtypes: lepidic (*p* < 0.001; Fig. 3B), acinar/papillary (*p* < 0.001; Fig. 3C), and solid/micropapillary (*p* = 0.028; Fig. 3D). Furthermore, subgroup analyses by tumor size indicated significant survival differences across clusters in  tumors measuring 1–2 cm (*p* < 0.001; Fig. 3F), 2–3 cm (*p* < 0.001; Fig. 3G), and 3–4 cm (*p* < 0.001; Fig. 3H). Within Cluster 2, patients at different stages (stage IA: *p* < 0.001; stage IB: *p* < 0.001; Fig. 3I, J) and those with VPI (*p* < 0.001; Fig. 3K) had poorer OS compared to the other two clusters. Multivariable Cox regression analysis further confirmed radiomic subtypes as an independent prognostic factor, with Cluster 2 displaying the highest mortality risk relative to Cluster 0 (HR = 15.71, 95% CI: 6.81–36.23, *p* < 0.001; Table 2).

**Fig. 4:**
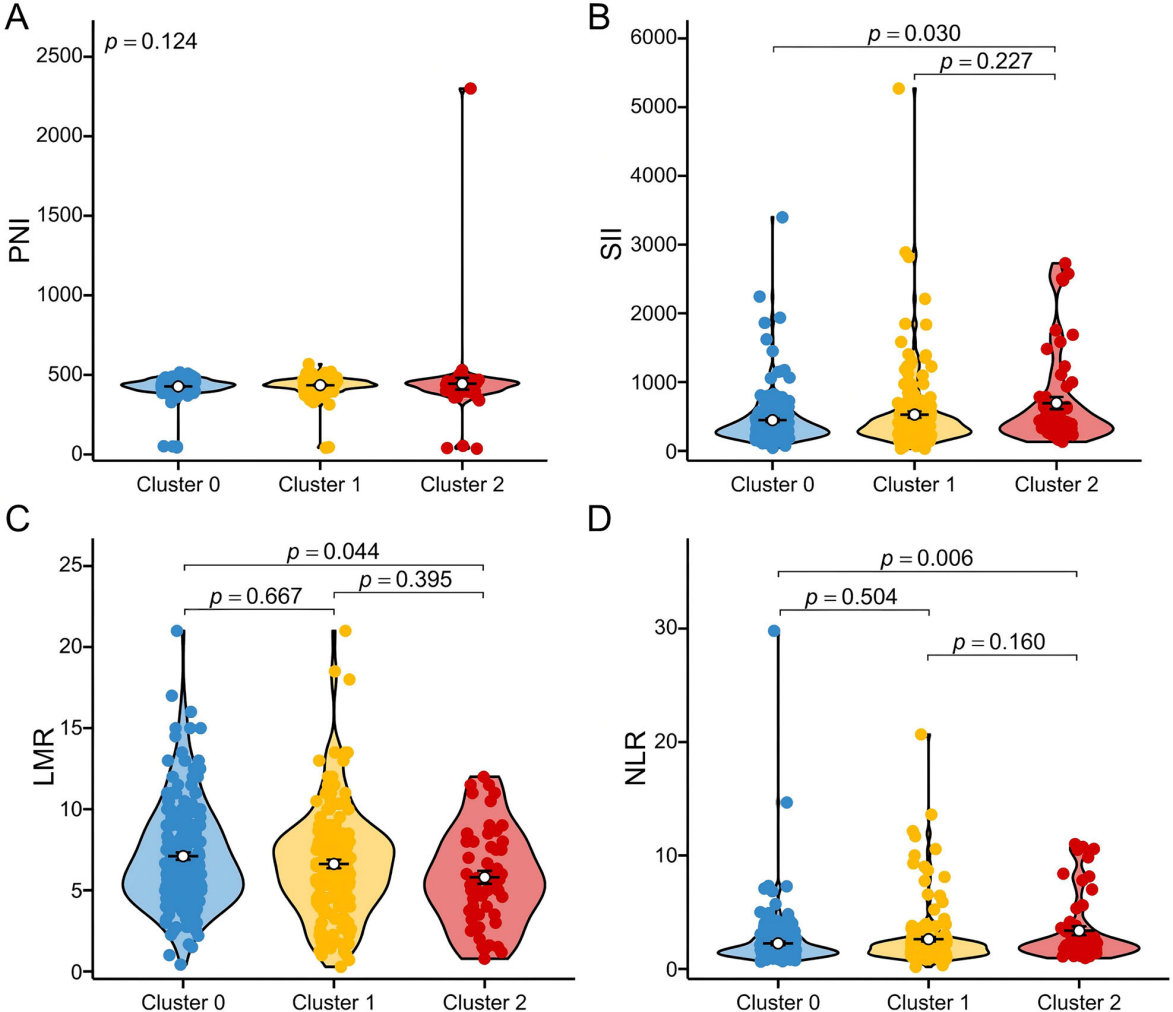
Nutritional-inflammatory characteristics of radiomic subtypes. **A** The distribution of PNI among the three clusters (overall *p* = 0.124). **B** The distribution of SII among the three clusters (Bonferroni-corrected pairwise comparisons: Cluster 2 vs Cluster 1, *p* = 0.030; Cluster 2 vs Cluster 1, *p* =0.227). **C** The distribution of LMR among the three clusters (Bonferroni-corrected pairwise comparisons: Cluster 1 vs Cluster 0, *p* = 0.667; Cluster 2 vs Cluster 1, *p* = 0.395; Cluster 2 vs Cluster 0, *p* = 0.044). **D** The distribution of NLR among the three clusters (Bonferroni-corrected pairwise comparisons: Cluster 1 vs Cluster 0, *p* = 0.504; Cluster 2 vs Cluster 1, *p* = 0.160; Cluster 2 vs Cluster 0, *p* = 0.006). PNI, prognostic nutritional index; SII, systemic immune-inflammation index; LMR, lymphocyte-to-monocyte ratio; NLR, neutrophil-to-lymphocyte ratio

**Table 2:** Multivariable Cox regression analyses for OS (*n* = 496)

Characteristic	HR (95% CI)	*p* value
Cluster		
Cluster 0	Reference	
Cluster 1	8.99 (3.82–21.15)	< 0.001
Cluster 2	15.71 (6.81–36.23)	< 0.001
Age	1.06 (1.03–1.08)	< 0.001
Gender		
Male	Reference	
Female	0.82 (0.53–1.27)	0.371
Pathological subtype		
Lepidic	Reference	
Acinar/papillary	0.83 (0.38–1.80)	0.633
Solid/micropapillary	6.01 (2.48–14.55)	< 0.001
Tumor size (cm)		
0–1	Reference	
1–2	1.04 (0.47–2.28)	0.929
2–3	1.42 (0.60–3.36)	0.421
3–4	2.80 (0.99–7.95)	0.053
TNM stage		
IA	Reference	
IB	0.45 (0.19–1.09)	0.078
VPI		
Absent	Reference	
Present	4.89 (2.35–10.18)	< 0.001

*HR* hazard ratio, *CI* confidence interval, *TNM stage* tumor-node-metastasis stage, *VPI* visceral pleural invasion

### Nutritional-inflammatory status of radiomic subtypes

To elucidate the relationship between radiomic subtypes and systemic nutritional-inflammatory status, we conducted an analysis of peripheral blood nutritional-inflammatory indicators from a cohort of 431 patients, all of whom were from the Shanghai Chest Hospital cohort. As illustrated in Fig. 4, global Kruskal–Wallis tests showed significant differences in SII, LMR, and NLR across the three radiomic subtypes, whereas PNI did not differ significantly (*p* = 0.124). In post-hoc pairwise Wilcoxon rank-sum tests with Bonferroni correction, SII and NLR were significantly higher and LMR was significantly lower in Cluster 2 compared with Cluster 0 (SII: Bonferroni-corrected *p* = 0.030; LMR: Bonferroni-corrected *p* = 0.044; NLR: Bonferroni-corrected *p* = 0.006), while no other pairwise comparisons reached statistical significance. Notably, Cluster 2, identified as the high-risk subtype, displayed a distinct inflammatory profile, characterized by elevated SII, decreased LMR, and increased NLR.

### Adjuvant chemotherapy benefit analysis based on radiomic subtypes

**Fig. 5:**
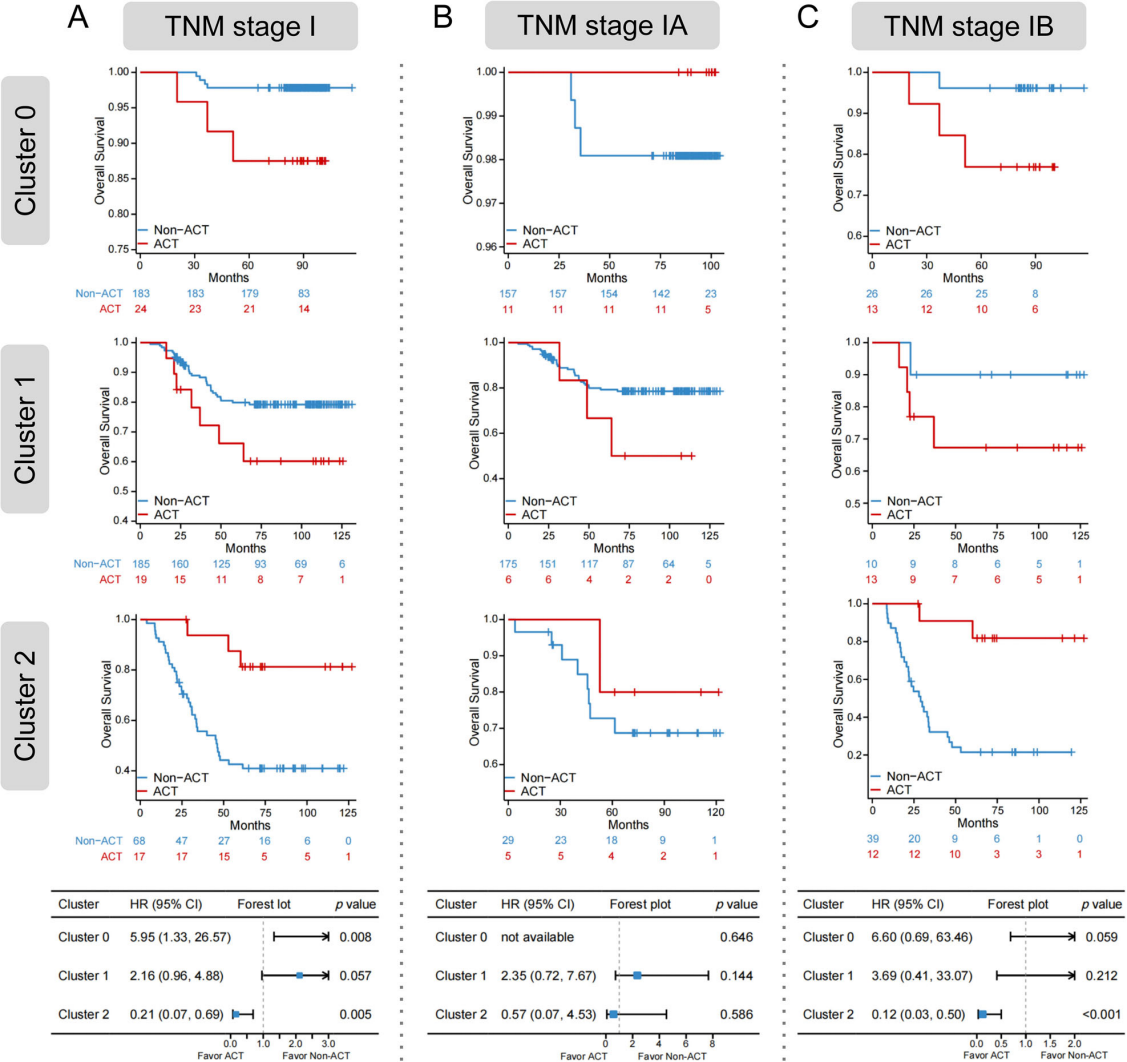
Kaplan–Meier curves according to treatment. **A** Predictive capacity for OS is stratified by treatment with ACT vs. non-ACT in patients with stage I (Cluster 2 vs Cluster 0: interaction *p* < 0.001). **B** Predictive capacity for OS is stratified by treatment with ACT vs non-ACT inpatients with stage IA (Cluster 2 vs Cluster 1: interaction *p* = 0.247). **C** Predictive capacity for OS is stratified by treatment with ACT vs. non-ACT in patients with stage IB (Cluster 2 vs Cluster 0: interaction *p* = 0.003). TNM stage, tumor-node-metastasis stage; ACT, adjuvant chemotherapy; HR, hazard ratio; CI, confidence interval

We further examined the predictive value of radiomic subtypes in determining the benefit of adjuvant chemotherapy across the three clusters. A significant interaction was observed between the different radiomic subtypes  in stage I patients and adjuvant chemotherapy (Cluster 2 vs Cluster 0: interaction *p* < 0.001; Fig. 5A), suggesting that radiomic subtypes may serve as predictors of chemotherapy benefit. Subgroup analyses were subsequently performed for stage IA and IB patients. In the stage IA subgroup, no significant interaction was detected (Cluster 2 vs  Cluster 1: interaction *p* = 0.247; Fig. 5B). By contrast, stage IB patients in Cluster 2 exhibited a significant OS benefit from adjuvant chemotherapy (Cluster 2 vs Cluster 0: interaction *p* = 0.003; Fig. 5C).

## Discussion

In this study, we developed an innovative CT-based radiomic subtype classification system using unsupervised clustering analysis, which demonstrated a significant association with the prognosis of early-stage LUAD and the nutritional-inflammatory status of patients. Specifically, Cluster 2 was associated with a heightened risk of mortality. Importantly, patients within Cluster 2 who underwent adjuvant chemotherapy experienced improved OS compared to those who did not receive such treatment, with this advantage being particularly evident in patients with stage IB disease. These findings suggest that our radiomic subtypes not only offer effective prognostic stratification for early-stage LUAD, but also help identify patients who are most likely to benefit from adjuvant chemotherapy.

To the best of our knowledge, this study is the first to demonstrate a significant association between radiomic subtypes and systemic nutritional-inflammatory status. The SII serves as a comprehensive measure that integrates the synergistic effects of neutrophils, lymphocytes, and platelets. The NLR reflects the balance between pro-tumor inflammation and anti-tumor immune responses [23]. Notably, elevated levels of SII and NLR indicate an immune imbalance characterized by neutrophil overactivation and lymphocyte dysfunction [24]. Extensive clinical evidence has demonstrated that increased SII and NLR are significantly associated with poorer OS in various solid tumors [23, 25, 26]. In our study, patients classified within Cluster 2 exhibited higher SII and NLR levels compared to those in Cluster 0, suggesting that this subtype is characterized by a pronounced inflammation-immune imbalance. This imbalance may be a critical factor contributing to tumor progression and poor prognosis [26]. Furthermore, the LMR serves as a pivotal indicator of the equilibrium between lymphocytes and monocytes. A decrease in LMR suggests a compromised anti-tumor immune response and the development of a pro-tumor microenvironment [27, 28]. In this study, the reduced LMR observed in patients within Cluster 2 indicates that this group exhibits a microenvironment characterized by suppressed lymphocyte function and immunosuppression mediated by tumor-associated macrophages. Taken together, these findings suggest that the inflammation-immune imbalance associated with Cluster 2 underlies its more aggressive behavior and poor prognosis. Additionally, they provide a potential biological rationale for tailoring treatment strategies in this high-risk subtype.

This study identified marked variations in responses to adjuvant chemotherapy across distinct radiomic subtypes. These results underscore the necessity of advancing beyond traditional TNM staging for patient stratification. Notably, patients classified within the low-risk Cluster 0 did not benefit from adjuvant chemotherapy and, furthermore, experienced significantly reduced OS following treatment. Conversely, patients in the high-risk Cluster 2 exhibited marked survival advantages from chemotherapy. From the standpoint of systemic nutritional-inflammatory and immunological characteristics, patients in Cluster 0 demonstrated a low systemic inflammatory burden, characterized by low SII, high LMR, and low NLR. This profile suggests the presence of a pre-existing effective anti-tumor immune response [29]. In this scenario, the non-specific cytotoxic effects of chemotherapy could potentially harm functional immune effector cells, particularly anti-tumor CD8+ T lymphocytes, thereby disrupting the otherwise effective immune microenvironment and ultimately offsetting the potential therapeutic benefits [30–32]. For high-risk patients in Cluster 2, we propose a dual mechanism underlying the benefits of chemotherapy. Firstly, it directly eradicates micro-metastatic lesions with a high risk of recurrence. Secondly, it may indirectly foster the recovery of anti-tumor immune function by depleting immunosuppressive cellular components [33]. Consequently, for Cluster 2 patients, the potent cytotoxic effects of chemotherapy appear to outweigh its detrimental impacts on immune function, thereby resulting in clinical benefits.

This study systematically investigated the significance of radiomic clustering in the assessment of tumor-related nutrition-inflammation, prognostic stratification, and treatment decision-making. However, several limitations must be acknowledged. Firstly, the retrospective design of the study introduces potential selection bias, which may affect reproducibility. Secondly, although our cohort was derived from two centers, all eligible patients were used for model development, and no independent external validation set was available. As a result, the data sources in this study are relatively limited, and the generalizability of the proposed radiomic subtypes should be interpreted with caution and confirmed in larger, prospectively collected cohorts from other institutions. Thirdly, although CT-based radiomic feature extraction is clinically practical and widely accessible, it is limited by the resolution and soft-tissue contrast, which may affect the accuracy of certain texture features. In addition, this retrospective multi-scanner cohort used non-harmonized acquisition and reconstruction settings (including both 512 × 512 and 1024 × 1024 matrix sizes), which may introduce residual variability in radiomic features despite IBSI-compliant extraction and feature standardization. Future investigations could benefit from incorporating multimodal imaging data, such as Positron Emission Tomography (PET)-CT, to enhance the comprehensiveness and precision of radiomic features. Lastly, this study is constrained by the absence of molecular biological evidence. Specifically, the lack of genetic mutation profiles and other molecular characteristics limits a deeper understanding of the biological mechanisms underlying the radiomic subtypes. Future research should integrate advanced technologies, such as single-cell sequencing and spatial transcriptomics, to elucidate the molecular underpinnings of distinct radiomic clusters, thereby enhancing the interpretability and robustness of the findings.

## Conclusion

In conclusion, this study successfully developed a CT-based radiomic subtype classification system for early-stage LUAD through unsupervised clustering. This system not only effectively stratified patients into distinct prognostic groups but also showed a clear association with systemic inflammatory-nutritional status. The high-risk subtype was associated with elevated inflammatory markers and exhibited a significant survival benefit from adjuvant chemotherapy, particularly in stage IB patients. These findings underscore the potential of radiomic subtypes to guide individualized postoperative treatment strategies.

## ELECTRONIC SUPPLEMENTARY MATERIAL

Supplementary information

## Data Availability

Any reasonable requests for access to available data underlying the results reported in this article will be considered. Such proposals should be submitted to the corresponding author.

## Abbreviations

ARI: Adjusted Rand Index
CI: Confidence interval
CT: Computed tomography
HR: Hazard ratio
HRCT: High-resolution CT
IASLC: International Association for the Study of Lung Cancer
IBSI: Image biomarker standardization initiative
IQR: Interquartile range
LMR: Lymphocyte-to-monocyte ratio
LUAD: Lung adenocarcinoma
LVI: Lymphovascular invasion
NLR: Neutrophil-to-lymphocyte ratio
OS: Overall survival
PNI: Prognostic nutritional index
SII: Systemic immune-inflammation index
TNM: Tumor-node-metastasis
VPI: Visceral pleural invasion
