# Supplementary material for: Radiomic subtypes predict survival and chemotherapy benefit in stage I lung adenocarcinoma: a multicenter study
Source: Insights Imaging. 2026 Mar 2;17:54. doi: 10.1186/s13244-026-02228-1 (PMC12953834; doi:10.1186/s13244-026-02228-1)

Radiomic Subtypes Predict Survival and Chemotherapy Benefit in Stage I Lung Adenocarcinoma: A Multicenter Study

ELECTRONIC SUPPLEMENTARY MATERIAL

Supplementary Table 1. Pairwise comparisons of baseline characteristics between clusters with Bonferroni-corrected *p* values.

Characteristic	Cluster 1 vs 0	Cluster 2 vs 0	Cluster 2 vs 1
Age (years)	0.310	0.348	**0.013**
Gender	0.221	**0.003**	0.211
Smoking history	1.000	**< 0.001**	**< 0.001**
Pathological subtype	**< 0.001**	**0.022**	**0.035**
Tumor size (cm)	**< 0.001**	**< 0.001**	**< 0.001**
TNM stage	0.115	**< 0.001**	**< 0.001**
VPI	1.000	**< 0.001**	**< 0.001**
LVI	0.262	0.114	1.000
ACT	1.000	0.198	0.054

TNM stage: Tumor-Node-Metastasis stage; VPI: Visceral pleural invasion; LVI: Lymphovascular invasion; ACT: Adjuvant chemotherapy.

Fig. S1 Flowchart of the patient selection process.

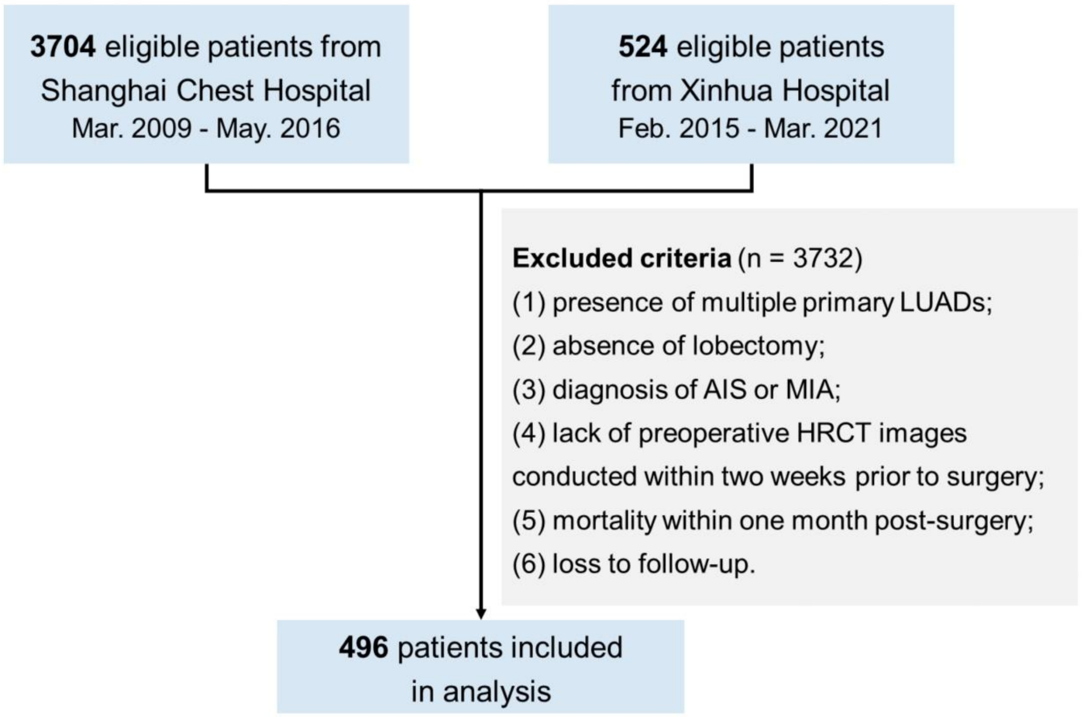

Supplement: Supplementary file 1 — Supplementary information [file 13244_2026_2228_MOESM1_ESM.pdf]
